# New Appliances and Things Medical

**Published:** 1899-12-23

**Authors:** 


### NEW APPLIANCES AND THINGS MEDICAL.

**[We shall be glad to receive, at our Office, 28 & 29, Southampton Street, Strand, London, W.C., from the manufacturers, specimens of all new preparations and appliances which may be brought out from time to time.]**

### BYNO-GLYCERO-PHOSPHATES.

(ALLEN AND HANBURYS, BETHNAL GREEN, LONDON, E.)

THE value of phosphates and hypophosphites in the treatment of wasting diseases, especially those which are associated with nervous exhaustion, has long been recognised, and many are the preparations now on the market which combine some form of these salts in union with glycerine. One of the most valuable of all these preparations is that which is known as Byno-glycero-phosphates, and which is a special solution of glycero-phosphates of iron, potash, magnesia, soda, and lime in Bynin liquid malt. The latter acts as a useful adjunct for the digestion of farinaceous foods, and is in itself of the nature of an assimilable food. The practical results following a course of treatment with Byno-phosphates cannot but prove satisfactory to the patient and physician alike; the treatment is simple, and cannot possibly have any harmful results.

### SPATEN VERSANDT BIER.

(GABRIEL SEDLMAYR, BRAUEREI ZUM SPATEN (MUNICH) ROSEBERY AVENUE, LONDON, E.C.)

WE have received two samples of this famous beer, namely, the dark and the pale, and can fully confirm the universal opinion that there is no finer or purer German beer to be obtained in the export trade. As is well known, Bavarian beer must by law be brewed exclusively from malt and hops, and Spaten beer forms no exception. Owing to its mildness and the absence of preservatives, there is some difficulty in keeping the beer in condition, whether bottled or in the cask. The firm appear to have taken ample precautions in their bottling department in this country to ensure this end. At the Spaten Beer Restaurant, Piccadily Circus, this beer may be obtained on draught, and in capital condition, or the bottled variety may be obtained by writing to the address given above. Orders under two dozen are in no cases supplied.

### MALTED NURSERY BISCUITS.

(W. HILL AND SON, 60, BISHOPSGATE STREET, LONDON.)

THESE biscuits are prepared from a farinaceous basis which has been submitted to Baron Liebig's malting process, whereby the starch is converted into various dextrines and sugars. By the application of iodine to the biscuit, the blue colouration which is given by starch was found to be entirely absent, and hence we may conclude the complete conversion of the original flour. This is a matter of great importance with infants, who are quite unable to digest unconverted starch and yet thrive on its malted equivalent. These biscuits seem popular with infants and young children, and although they may be advantageously used as directed, namely, after soaking in water or milk, they may be equally well employed as an experimental medium on which to try the newly-cut tooth.

### SWINBORNE'S PATENT REFINED ISINGLASS.

(G. P. SWINBORNE, 33 AND 34, ST. ANDREW'S HILL, QUEEN VICTORIA STREET, LONDON, E.C.)

THIS isinglass, which is prepared according to Swinborne's patent process, is a remarkably pure article, the purity being obtained by very simple means—by the solvent action of water without the use of chemical re-agents, or bleaching substances. The undoubted purity of Swinborne's isinglass renders it particularly valuable for invalid cookery, especially in the preparation of clear soups, meat, fruit, wine, and other jellies.

